# Evolution-guided adaptation of an adenylation domain substrate specificity to an unusual amino acid

**DOI:** 10.1371/journal.pone.0189684

**Published:** 2017-12-14

**Authors:** Simon Vobruba, Stanislav Kadlcik, Radek Gazak, Jiri Janata

**Affiliations:** Institute of Microbiology of the Czech Academy of Sciences, Prague, Czech Republic; Universidade Nova de Lisboa, PORTUGAL

## Abstract

Adenylation domains CcbC and LmbC control the specific incorporation of amino acid precursors in the biosynthesis of lincosamide antibiotics celesticetin and lincomycin. Both proteins originate from a common L-proline-specific ancestor, but LmbC was evolutionary adapted to use an unusual substrate, (2*S*,4*R*)-4-propyl-proline (PPL). Using site-directed mutagenesis of the LmbC substrate binding pocket and an ATP-[^32^P]PPi exchange assay, three residues, G308, A207 and L246, were identified as crucial for the PPL activation, presumably forming together a channel of a proper size, shape and hydrophobicity to accommodate the propyl side chain of PPL. Subsequently, we experimentally simulated the molecular evolution leading from L-proline-specific substrate binding pocket to the PPL-specific LmbC. The mere change of three amino acid residues in originally strictly L-proline-specific CcbC switched its substrate specificity to prefer PPL and even synthetic alkyl-L-proline derivatives with prolonged side chain. This is the first time that such a comparative study provided an evidence of the evolutionary relevant adaptation of the adenylation domain substrate binding pocket to a new sterically different substrate by a few point mutations. The herein experimentally simulated rearrangement of the substrate binding pocket seems to be the general principle of the *de novo* genesis of adenylation domains’ unusual substrate specificities. However, to keep the overall natural catalytic efficiency of the enzyme, a more comprehensive rearrangement of the whole protein would probably be employed within natural evolution process.

## Introduction

**Fig 1:**
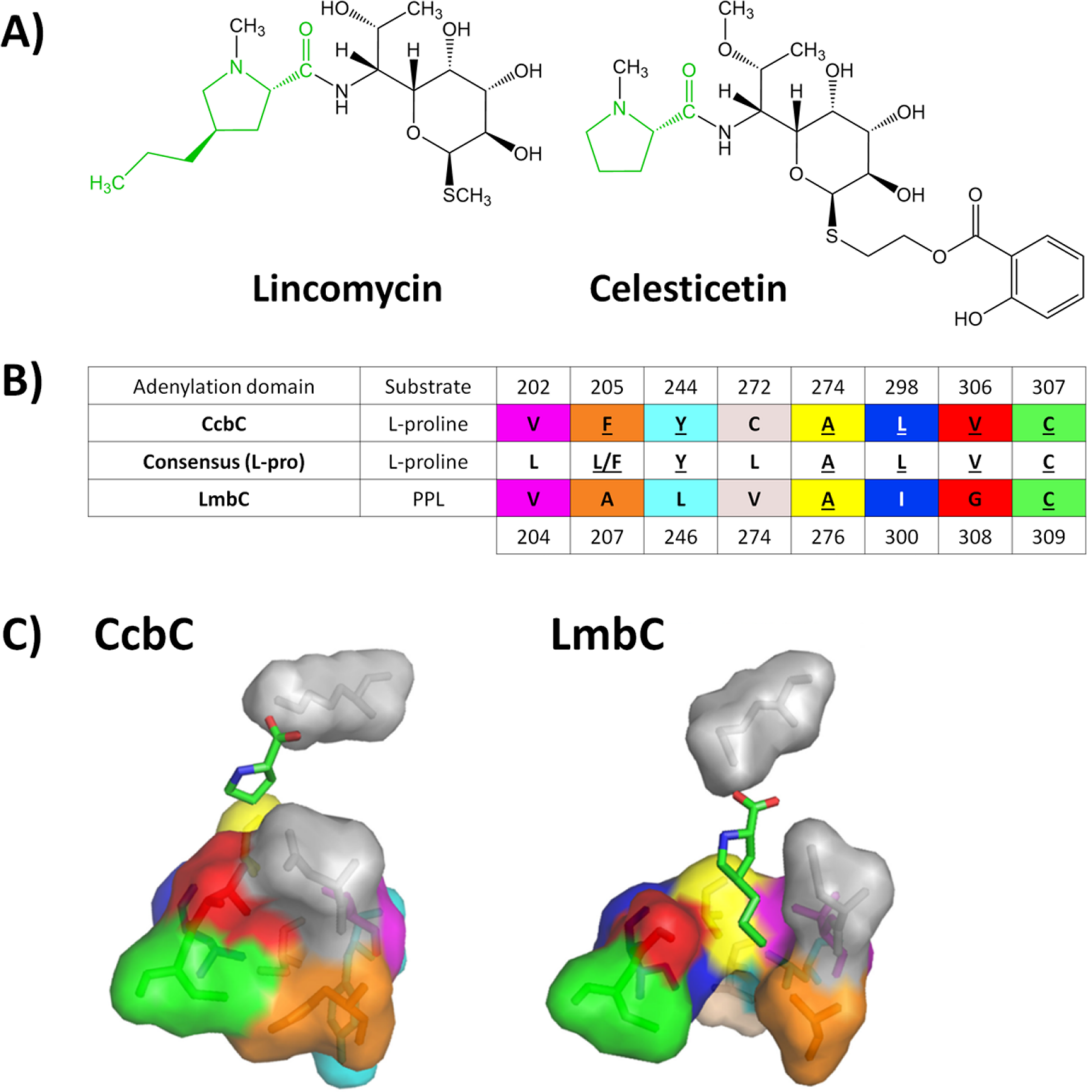
Comparison of the nonribosomal codes of CcbC and LmbC substrate binding pockets (SBPs). **A)** Structures of lincomycin and celesticetin. Amino acid precursors activated by adenylation domains (A-domains) are indicated in green. **B)** Pattern of eight variable amino acid residues of CcbC and LmbC nonribosomal codes. The highly conserved D and K residues at the boundaries of the nonribosomal codes are omitted. Amino acid residues are numbered according to CcbC (first row) and LmbC (last row). The consensus code of the stand-alone L-proline-specific A-domains is shown in the middle row [3]. The residues in LmbC and CcbC SBPs, which correspond to the consensus, are underlined. Colours correspond to the individual amino acid residues in the model of CcbC/LmbC SBPs (C). **C)** Homology models of the CcbC SBP with L-proline and the LmbC SBP with PPL [3].

Lincosamides are a small but clinically important group of antibiotics consisting of only two compounds with a characterised biosynthetic gene cluster, lincomycin and celesticetin (Fig 1A), produced by *Streptomyces lincolnensis* and *Streptomyces caelestis*, respectively. The crucial step of lincosamide biosynthesis is the condensation of amino sugar and amino acid precursors via an amide bond [1]. While the amino sugar precursor of both natural lincosamides is identical, the biosynthetic origin and availability of the incorporated amino acid (green in Fig 1A) differ. The celesticetin precursor, L-proline, is a regular component of the cellular proteinogenic amino acid pool, while the lincomycin precursor is an unusual alkyl-L-proline derivative (APD), (2*S*,4*R*)-4-propyl-proline (PPL), a product of the specialised biosynthetic pathway originated from L-tyrosine [2].

The three-carbon (3C) propyl side chain of the proline moiety positively affects the antibacterial activity of lincomycin compared with celesticetin [4]. Recently prepared derivatives of celesticetin with incorporated PPL instead of L-proline exhibited even higher antibacterial activity than both natural lincosamides [5]. Moreover, synthetically- or mutasynthetically-prepared derivatives of lincomycin with a prolonged alkyl side chain (4C, 5C) exhibit increased antibacterial and even significant antiplasmodial activities [4,6].

The condensation reaction in lincosamide biosynthesis is catalysed by a multimeric enzyme composed of a unique condensation protein and stand-alone nonribosomal peptide synthetases (NRPS) components—the adenylation domain (A-domain) and a carrier protein. The A-domain recognises the amino acid substrate and activates its carboxyl functional group by binding of adenosine monophosphate [3]. The activated amino acid precursor is subsequently attached to the carrier protein [7] and condensed with the activated amino sugar precursor [1]. In lincomycin biosynthesis, the PPL precursor is specifically recognised and activated by A-domain LmbC, while the homologous protein CcbC from celesticetin biosynthesis is strictly L-proline-specific [3]. The substrate specificity of the A-domain thus determines which amino acid will be incorporated in the molecule of the resulting lincosamide.

Phylogenetic analysis of CcbC and LmbC revealed that they both belong to the subfamily of stand-alone L-proline-specific A-domains. Their sequence identity to these L-proline-specific A-domains from biosyntheses of various natural products ranges from 33.0 to 39.7% [3]. Nevertheless, the CcbC/LmbC mutual 55.7% identity [7,8] significantly exceeds this level, suggesting their direct evolution from a common L-proline-specific ancestor. It makes this pair a suitable experimental model for the study of molecular evolution of A-domain substrate specificity.

Substrate specificity of the A-domain is determined by a “nonribosomal code” consisting of 10 amino acid residues that create a substrate binding pocket (SBP). Two SBP residues (lysine and glutamate) interacting with the carboxy- and amino-group of substrate, respectively, are conserved in all amino acid-activating A-domains. The remaining eight variable residues are supposed to determine substrate specificity [9–11]. The nonribosomal code of LmbC differs from that of CcbC in five of the eight variable amino acid residues (Fig 1B), likely as a result of its adaptation to use the unusual PPL precursor. Homology models of LmbC/CcbC SBPs with PPL or L-proline substrate, respectively, show that those differences in nonribosomal codes probably result in differences in the overall size, shape and hydrophobicity between both SBPs (Fig 1C) [3]. The modelled CcbC SBP has a smaller cavity, where the substrate is in contact with only three variable residues of the nonribosomal code—V202, A274 and V306. This binding site thus appears to be too small to accommodate the alkyl side chain of PPL. In contrast, in the homology model of the LmbC SBP, a hydrophobic channel accommodating the alkyl side chain of PPL has been predicted [3].

A-domains that activate either proteinogenic or, even more often, unusual amino acids are an indispensable part of the biosynthesis of the large portion of existing natural compounds. Here, we used a unique system of two functionally characterised and evolutionary closely related stand-alone A-domains, LmbC and CcbC, and attempted to simulate the process of the molecular evolution of the substrate specificity of the L-proline-specific A-domain to activate the unusual APD.

## Materials and methods

### Materials

(2*S*,4*R*)-4-ethyl-proline (EPL), (2*S*,4*R*)-4-propyl-proline (PPL), (2*S*,4*R*)-4-butyl-proline (BuPL) and (2*S*,4*R*)-4-pentyl-proline (PePL) were prepared according to a previously described procedure [3,6]. Other chemicals were purchased from Sigma-Aldrich (Germany) unless otherwise stated.

C-terminal His_8_-tagged LmbC and LmbC G308V and N-terminal His_6_-tagged CcbC were produced as described previously [3] from vectors plmbC1, plmbC4 and pccbC, respectively.

### Site-directed mutagenesis and construction of expression vectors

Site-directed mutagenesis of *lmbC* was performed using the vector plmbC3 [3] as a template and the QuickChange Site-Directed Mutagenesis Kit (Stratagene, USA) as described previously for the preparation of LmbC G308V [3]. Site-directed mutagenesis of *ccbC* was performed analogously to the mutagenesis of *lmbC*: first, the *ccbC* gene was excised via the *Nde*I and *Hind*III restriction sites from the pccbC vector and inserted into a pJAKO cloning vector [12] using the same restriction sites. Next, the resulting pccbC2 plasmid was used as a template for the *in vitro* site-directed mutagenesis of *ccbC*. Primers used for site-directed mutagenesis are listed in S1 Table. Multiple mutations were prepared by repeating the site-directed mutagenesis protocol using the already mutated plmbC3 or pccbC2 as a template.

The mutated genes *lmbC* (excised via *Nde*I and *Xho*I restriction sites) and *ccbC* (excised via *Nde*I and *Hind*III restriction sites) were inserted into expression vectors pET42b and pET28b, respectively. The open reading frames were confirmed by sequencing. The resulting vectors were used for the production of LmbC and CcbC mutant proteins with a C-terminal His_8_-tag and an N-terminal His_6_-tag, respectively.

### Preparation of the chimeric adenylation domain

Outer portions of the *ccbC* gene were amplified from the plasmid pccbC2 using the primer pair CcbC1_for and CcbC1_rev and primer pair CcbC2_for and CcbC2_rev (S2 Table). The central part of the *lmbC* gene, coding for amino acid residues 173 to 315, was amplified from the plasmid plmbC3 using the primers LmbC1_for and LmbC1_rev (S2 Table). The outer and central parts were fused by PCR using primers CcbC1_for and CcbC2_rev. The chimeric *ccbC* gene was inserted into the pET28b expression vector via *Nde*I and *Hind*III restriction sites. The resulting plasmid was used for production of the N-terminal His_6_-tagged protein. The open reading frame of the chimeric gene was confirmed by sequencing.

### Heterologous production and purification of proteins

All proteins were heterologously produced in *Escherichia coli* BL21 (DE3) as described previously [3] at a postinduction temperature of 17°C for 20 hours. Protein purification was performed according to a previously described method [3]. The CcbC, CcbC mutants and chimeric CcbC proteins were washed on a column with TS-8 buffer containing 50 mM imidazole and the LmbC and LmbC mutants were washed with TS-8 buffer containing 100 mM imidazole. All proteins were eluted with TS-8 buffer containing 250 mM imidazole. The concentration of purified proteins was determined spectrophotometrically.

### Enzyme activity assay

The A-domains were biochemically characterised using an ATP-[^32^P]PPi exchange assay—the amino acid-dependent exchange of radioactivity from [^32^P]-labelled PPi into ATP. This standard method was previously used for the characterisation of other stand-alone A-domains [13–15]. The enzyme activity assay was conducted as described previously [3] to ensure the comparability of results. The linearity of reaction velocity during the 30-minute testing range was confirmed. Negative control reactions were conducted by excluding substrate. The kinetic parameters were determined by non-linear regression using the programme KaleidaGraph 4.5.2.

## Results and discussion

### LmbC SBP mutagenesis: Detection of residues affecting the affinity for PPL

We assessed the impact of amino acid residues of the LmbC SBP on its preference for PPL over L-proline. Amino acid residues of the LmbC nonribosomal code, which differ from corresponding residues of the CcbC nonribosomal code (Fig 1B), were individually replaced by their CcbC counterpart. His-tagged forms of the mutated A-domains were heterologously produced and purified as described in the experimental section. Their activities were determined using the ATP-[^32^P]PPi exchange assay. The kinetic parameters of LmbC and LmbC single mutants for PPL and L-proline are summarised in Table 1 and the Michaelis-Menten plots in S1 Fig.

**Table 1:** Kinetic parameters of LmbC and LmbC single mutants for PPL and L-proline substrates.

	PPL			L-proline		
Adenylation domain	K_m_ [mM]	k_cat_ [min^-1^]	k_cat_/K_m_ [mM^-1^ min^-1^]	K_m_ [mM]	k_cat_ [min^-1^]	k_cat_/K_m_ [mM^-1^ min^-1^]
LmbC ^[a]^	0.28 ± 0.03	33 ± 1	120	480 ± 70	20 ± 1	0.042
LmbC I300L	0.24 ± 0.009	45 ± 0.5	185	190 ± 30	23 ± 2	0.12
LmbC V274C	0.39 ± 0.04	39 ± 1	100	250 ± 30	19 ± 1	0.07
LmbC G308V ^[a]^	5.8 ± 0.6	0.39 ± 0.02	0.07	240 ± 20	4.9 ± 0.2	0.02
LmbC A207F	8.6 ± 1	4.4 ± 0.3	0.51	380 ± 40	2.3 ± 0.1	0.006
LmbC L246Y	33 ± 6	0.37 ± 0.03	0.011	54 ± 2	13 ± 0.2	0.24

[a] The previously characterized form [3], re-measured in the frame of the new experiments.

PPL—(2*S*,4*R*)-4-propyl-proline. The error values indicate the standard error.

LmbC single mutants can be divided into two groups according to their activity in reactions with PPL. The first group includes mutants LmbC I300L and LmbC V274C, whose kinetic parameters only slightly differ from LmbC. Residues in these positions may have been subject of a random mutation during the evolution of LmbC, with minimal influence on the final PPL specificity. Examples of variability in one or two residues of the nonribosomal code of related stand-alone proline-specific A-domains were reported previously [16,17].

The remaining three mutations significantly affected the LmbC acceptance of PPL. From the comparison of K_m_ values of LmbC G308V and LmbC A207F, it is apparent that the affinity of these mutants for PPL was more than 10 times lower in contrast to LmbC. The LmbC residues (G308 and A207) with no or minimal side chain, respectively, likely contribute to the formation of the channel of the proper shape and size to accommodate the propyl side chain of PPL (Fig 1C, red and orange). Conversely, the function of residue L246, which was experimentally documented to have the highest impact on the affinity to PPL (Fig 1C, light blue), cannot be fully explained by homology models, except for the possible adjustment of hydrophobicity of the channel in the SBP [3]. However, both the affinity and catalytic rate constant of LmbC L246Y for PPL were two orders lower compared with LmbC characteristics. The L246Y mutation is also the only one that decreases the Km value of LmbC for L-proline by an order. We can only speculate that the tyrosine large planar side chain may stabilise the SBP and makes it more compact and suitable for the binding of L-proline, similar to the CcbC SBP. It is also possible that the corresponding tyrosine residue Y244 in CcbC interacts with F205 either by π-π stacking or simply by steric effects to better accommodate the L-proline, which is not the case for LmbC L246Y, where A207 (conform to F205 of CcbC) is unable to delineate by a similar way the steric orientation of the artificially introduced Y246. Our results together suggest that L246Y may fulfil an important role in the LmbC SBP, but it probably cannot be elucidated without the crystal structures of LmbC/CcbC proteins.

The single mutations of three abovementioned important residues negatively influenced the LmbC affinity for PPL, and in addition their combination completely abolished its activation, confirming the significance of these residues, as summarised in S3 Table and S1 Fig. The channel that accommodates the propyl side chain was probably completely blocked in the LmbC double and triple mutants. It should be noted that all these mutants were still active in reactions with L-proline, indicating that the proper protein fold was at least partially preserved.

In summary, these experiments evaluated the previously designed homology models of CcbC/LmbC SBPs and revealed three amino acid residues (G308, A207 and L246) in the LmbC SBP that are significant for LmbC affinity for PPL. These residues together likely contribute to the formation of a channel of a proper size, shape and hydrophobicity to accommodate the propyl side chain of PPL.

### CcbC mutagenesis: Verification of the evolutionary adaptation of the A-domain SBP to accommodate unusual PPL

After elucidation of the key LmbC residues affecting the acceptance of PPL, we used CcbC to experimentally verify the evolutionary adaptation of the L-proline-specific A-domain substrate specificity to prefer PPL. Residues in the CcbC SBP located in the corresponding positions to the three abovementioned significant LmbC residues were replaced by them. All CcbC mutants included a mutation of the essential residue V306, which interferes with proximal atoms of the substrate’s alkyl side chain and sterically hinders its accommodation (Fig 1C, red). It was subsequently combined with mutations F205A and/or Y244L, which are localised deeper in the alkyl side chain-accommodating channel, resulting in double and triple mutants.

The affinity of all tested CcbC mutants for L-proline and various APDs is summarised in Table 2 and the Michaelis-Menten plots in S2 Fig. For all of them, the K_m_ for L-proline increased by 2–3 orders when compared with CcbC. The inhibition of an efficient L-proline activation is a necessary part of the adaptation to the PPL substrate, because of the presence of L-proline in the cellular proteinogenic amino acid pool. The combination of the two mutations (V306G and F205A) even reduced the affinity for L-proline to the K_m_ value similar to that of LmbC.

**Table 2:** K_m_ values of CcbC, CcbC mutants and LmbC in reaction with various substrates.

	K_m_ [mM]				
Adenylation domain	L-proline	EPL	PPL	BuPL	PePL
CcbC ^[a]^	0.36 ± 0.03	NA	NA	NT	NT
CcbC V306G	37 ± 2	NA	NA	NT	NT
CcbC V306G + F205A	670 ± 180	NA	NA	NT	NT
CcbC V306G + Y244L	86 ± 6	27 ± 3	24 ± 3	LA	NA
CcbC V306G + F205A + Y244L	670 ± 60	31 ± 7	6.4 ± 1	5.8 ± 1	2.5 ± 1
LmbC ^[a]^	480 ± 70	6.4 ± 0.3	0.28 ± 0.03	0.12 ± 0.006	0.06 ± 0.003

[a] The previously characterized form [3], re-measured in the frame of the new experiments.

EPL—(2*S*,4*R*)-4-ethyl-proline; PPL—(2*S*,4*R*)-4-propyl-proline; BuPL—(2*S*,4*R*)-4-butyl-proline and PePL—(2*S*,4*R*)-4-pentyl-proline.

NA–tested, no detectable activity. NT–not tested. LA–low detected activity, not possible to determine the kinetic parameters. The error values indicate the standard error.

Another CcbC double mutant (V306G + Y244L) exhibits modified substrate specificity and is also capable of activating APDs with 2C or 3C side chains. However, the natural substrate of LmbC, PPL, is strongly preferred over L-proline only by the CcbC triple mutant with the additional F205A mutation. Based on the homology model, this mutation likely facilitates the accommodation of distal atoms of the PPL side chain into the channel in the SBP. In accordance, the triple mutant also activates the synthetic L-proline derivatives with prolonged alkyl side chains, (2*S*,4*R*)-4-butyl-proline (BuPL) and (2*S*,4*R*)-4-pentyl-proline (PePL), with K_m_ values even lower than those for PPL. This decreasing trend of K_m_ values from L-proline to PePL mimics the substrate preference of LmbC [3]. It should be mentioned that the CcbC triple mutant retains 99.4% identity with the strictly L-proline-specific CcbC but only 56.2% identity with the PPL-preferring LmbC. In other words, there are 224 remaining differences (214 substitutions and 10 insertions/deletions) between LmbC and CcbC triple mutant with identical substrate specificity patterns. Our results show that so minor modification of the overall primary structure as these three substitutions in the SBP of the L-proline-specific A-domain is sufficient to simulate the evolutionary adaptation of its substrate specificity to a new unusual substrate.

Site-directed mutagenesis, guided by the nonribosomal code, was previously used in several studies to alter A-domain substrate specificity [18–22]. Nevertheless, in any of these experiments, such a conclusively evolutionary close but substrate specificity divergent pair such as CcbC/LmbC has not been studied. This is the first time that such a comparative study provided an evidence of the evolutionary adaptation of the A-domain substrate specificity to a new sterically different substrate by a few point mutations.

At the molecular level, this evolutionary shift is probably caused by a dramatic rearrangement of the SBP, specifically by formation of a hydrophobic channel accommodating the alkyl side chain of the substrate, while binding of the L-proline without any alkyl side chain is disadvantaged. The formation of a channel in the SBP accommodating a prolonged alkyl side chain was recently reported in a comparative study of another pair of related A-domains differing in substrate specificity. The incednine A-domain has a shallow SBP, where the bulky L220 residue prevents the incorporation of a substrate with a longer side chain. In contrast, the cremimycin A-domain possesses a smaller residue, G220, at the corresponding position, allowing the tunnel to extend over the position of G220 and accommodate the substrate’s side chain [23].

In contrast to the shift in substrate specificity, the overall catalytic efficiency of PPL-activating CcbC double and triple mutants is far from the parameters of LmbC. As shown in Table 3, the catalytic rate constant, and thus the overall catalytic efficiency, is significantly lower compared to LmbC. It can be at least partially explained by nonselective worsening of the overall catalytic efficiency of CcbC mutants, as the catalytic rate constant for L-proline is also reduced (see Table 3 and S4 Table for other tested CcbC mutants). The nonselective worsening of the overall catalytic efficiency is, however, a common consequence of multiple artificial changes in natural proteins [18,19,21,24–26].

**Table 3:** Kinetic parameters of LmbC, CcbC and selected CcbC mutants for various substrates.

Adenylation domain	Substrate	K_m_ [mM]	k_cat_ [min^-1^]	k_cat_/K_m_ [mM^-1^ min^-1^]
CcbC ^[a]^	L-proline	0.36 ± 0.03	55 ± 1	153
CcbC V306G + Y244L	L-proline	86 ± 6	2 ± 0.05	0.025
CcbC V306G + F205A + Y244L	L-proline	670 ± 60	6 ± 0.3	0.009
LmbC ^[a]^	L-proline	480 ± 70	20 ± 1	0.042
CcbC V306G + Y244L	PPL	24 ± 3	0.06 ± 0.003	0.0026
CcbC V306G + F205A + Y244L	PPL	6.4 ± 1	0.02 ± 0.001	0.003
LmbC ^[a]^	PPL	0.28 ± 0.03	33 ± 1	120
CcbC V306G + F205A + Y244L	PePL	2.5 ± 1	0.025 ± 0.003	0.01
LmbC ^[a]^	PePL	0.06 ± 0.003	55 ± 0.8	920

[a] The previously characterized form [3], re-measured in the frame of the new experiments.

PPL—(2*S*,4*R*)-4-propyl-proline; PePL—(2*S*,4*R*)-4-pentyl-proline. The error values indicate the standard error.

Moreover, in contrast to substrate specificity, the overall catalytic efficiency is also affected by the amino acid residues neighbouring the SBP and the entire tertiary structure of the A-domain [18,24]. We suggest that the worse overall catalytic efficiency of CcbC mutants for PPL may be the result of incompatibility between artificially-changed residues in the SBP and some of the hundreds of residues changed outside of the SBP during the separated evolution of CcbC and LmbC proteins from their common L-proline-specific ancestor.

In addition to the evolutionary significance described herein, this type of studies also has an application potential. More than a hundred hybrid lincosamide compounds were recently prepared *in vitro* using the combination of enzymatic activities from celesticetin and lincomycin biosynthesis [5]. Those that combine the incorporation of the lincomycin-specific PPL precursor together with the salicylate unit, which is specific for celesticetin, exhibited even higher antibacterial activity than the clinically important lincomycin. Based on the knowledge of salicylate attachment in celesticetin biosynthesis [5,27–29], a celesticetin-producing strain with genetically engineered CcbC to accept PPL or APD with prolonged side chains could be used for the mutasynthetic preparation of the most potent lincosamide compounds, even with significant antimalarial activity [4–6]. Nevertheless, the fully active enzyme is necessary for these practical purposes. The approaches used to increase the overall catalytic efficiency should take into consideration the entire protein sequence. These methods resemble recombination, an evolutionary mechanism described in modular NRPS A-domains [30–33]. Artificial recombination has been successfully used to prepare chimeric proteins from the modular NRPS A-domains in hormaomycin biosynthesis [32]. Using this approach we prepared the soluble chimeric LmbC/CcbC protein, nevertheless it was inactive in reactions with both L-proline and PPL.

### Evolutionary impact of the lincosamide model in the context of other APD activating A-domains

Adaptation of the L-proline-specific A-domain to use an unusual PPL precursor was an important milestone in the molecular evolution of lincosamide biosynthesis, resulting in the production of the more efficient antibiotic, lincomycin. Analogous scenario i.e. the evolution of metabolites involving an APD moiety instead of the L-proline emerged several times in nature. APD precursors nearly identical to PPL are incorporated into anticancer pyrrolo[2,1-c][1,4]benzodiazepines (PBDs; S3 Fig) [34,35] and the bacterial signalling molecule, hormaomycin (S3 Fig) [32]. Accordingly, the biosynthetic pathways of all these APD containing compounds share nearly identical set of 5–6 enzymes encoded by APD biosynthetic gene cluster spread by the mechanism of horizontal gene transfer [2, 36–39].

In contrast to the common origin of the APD biosynthetic genes, phylogenetic analysis convincingly documented that the relevant APD specific A-domains evolved independently from different ancestors in the biosynthesis of PBDs, hormaomycin and lincomycin [3] (updated in S4 Fig). Nevertheless, in all three cases, APD-specific A-domains arose from L-proline-specific ancestors. We suggest that their adaptation to a new unusual amino acid substrate occurred by an identical molecular mechanism as the adaptation of LmbC, by point mutations in the SBP of an L-proline-specific ancestor. It can be demonstrated by the example of the SibD A-domain from the biosynthesis of PBD sibiromycin. The variable residues of its nonribosomal code (VMFYTALV) differ from the consensus code of related L-proline-specific modular NRPS A-domains (VQ(F/Y)IAHVV) in five underlined residues. It resembles the dramatic rearrangement of the SBP in A-domains from lincosamide biosynthesis.

Because compounds with incorporated unusual amino acid precursors form a large portion of all occurring natural products, the genesis of substrate specificity of the corresponding A-domains is a topic of high general significance. Here we documented this process on a model of molecular evolution of a pair of stand-alone A-domains. Even though the evolutionary mechanism of recombination has been described for more frequent A-domains of modular NRPSs [30–33], this mechanism can only elucidate the emergence of new combinations of incorporated amino acid units, but not the genesis of unusual substrate specificity *de novo*. The presented SBP rearrangement thus seems to be the general principle for the molecular evolution of both groups of A-domains.

## Supporting information

S1 Fig Comparison of LmbC and the various LmbC mutants’ reaction kinetics for various substrates. The tested proteins and substrates are written above each graph. All reactions were performed in triplicate. The error bars indicate the standard deviation. The reaction velocity is expressed as the amount of radioactive ATP (mM) produced per minut. Reaction conditions are described in the experimental section.
(TIF)

S2 Fig Comparison of CcbC and the various CcbC mutants’ reaction kinetics for various substrates. The tested proteins and substrates are written above each graph. All reactions were performed in triplicate. The error bars indicate the standard deviation. The reaction velocity is expressed as the amount of radioactive ATP (mM) produced per minut. Reaction conditions are described in the experimental section.
(TIF)

S3 Fig Structures of selected pyrrolo[2,1-*c*][1,4]benzodiazepines, hormaomycin and their amino acid precursors. Amino acid precursors activated by appropriate A-domains are in the first column.DH-EPL—4-ethylidene-L-proline, DH-PPL—4-propylidene-L-proline, (4-Pe)Pro—4-propenyl-L-proline.
(TIFF)

S4 Fig Phylogenetic relationships of A-domains specific for L-proline or its derivatives. A rooted, maximum likelihood phylogenetic tree was constructed based on the full length amino acid sequences of representative stand-alone A-domains and excised sequences of representative modular NRPS A-domains. Bootstrap values (100 replicates) above 50% are indicated at the nodes. Number in parentheses behind the name of respective NRPS denotes the number of the module in NRPS protein chain, if relevant. The substrate of each A-domain is indicated next to its name. The substrates include L-proline (Pro), L-proline derivatives with two carbon side chain (Pro2C), and L-proline derivatives with three carbon side chain (Pro3C). A-domains specific for Pro2C or Pro3C substrates are highlighted in blue. Their closely related A-domains specific for L-proline are highlighted in grey. The phylogenetic analysis separated A-domains into two clades. Stand-alone A-domains that all, except LmbC, activate L-proline are above the dotted line. Modular NRPS A-domains are below the line, where are the APD activating A-domains split into two branches (Por21, Orf22, SibD, TomB from the biosynthesis of representative PBDs and HrmP(3) from the biosynthesis of hormaomycin). The GenBank accession numbers of stand-alone A-domains are IdmJ–ACN6998.1, CalN2 –AEH42484.1, NgnN4 –AEI59690.1, CouN4 –AAG29789.1, DkxA–CAQ34914.1, Bmp4 –AKJ75110.1, PigI–CAH55654.1, MarM–AHF22853.1, RedM–CAA16182.1, LmbC–ABX00600.1, CcbC–ADB03652.1, Leu5 –ADZ24989, AnaC–ACR33075.1, HrmK–AEH41789.1.The GenBank accession numbers of modular NRPS A-domains are Por21 –AEA29644.1, Orf22 –ABW71853.1, SibD–ACN39727.1, TomB–ACN39015.1, NpsB–CDG76959.1, MchC(2)–CAG29032.1, CipA(2)–AHZ34238.1, NosD(2)–AAF17281.1, PuwA(2)–AIW82277.1, GrsB(1)–BAA06146.1, ItuB(4)–BAB69699.1, HrmP(3)–AEH41794.1, LpmD(2)–AEG64698.1, ACMSIII(1)–CCO61885.1.
(TIF)

S1 Table Primers used for site-directed mutagenesis. (DOCX)

S2 Table Primers used for preparation of the chimeric *ccbC* gene. (DOCX)

S3 Table Kinetic parameters of LmbC and LmbC mutants for L-proline and PPL substrates. (DOCX)

S4 Table Kinetic parameters of the remaining CcbC mutants for various substrates. (DOCX)
