# Supplementary figures and images for: Evolution-guided adaptation of an adenylation domain substrate specificity to an unusual amino acid
Source: PLoS One. 2017 Dec 14;12(12):e0189684. doi: 10.1371/journal.pone.0189684 (PMC5730197; doi:10.1371/journal.pone.0189684)

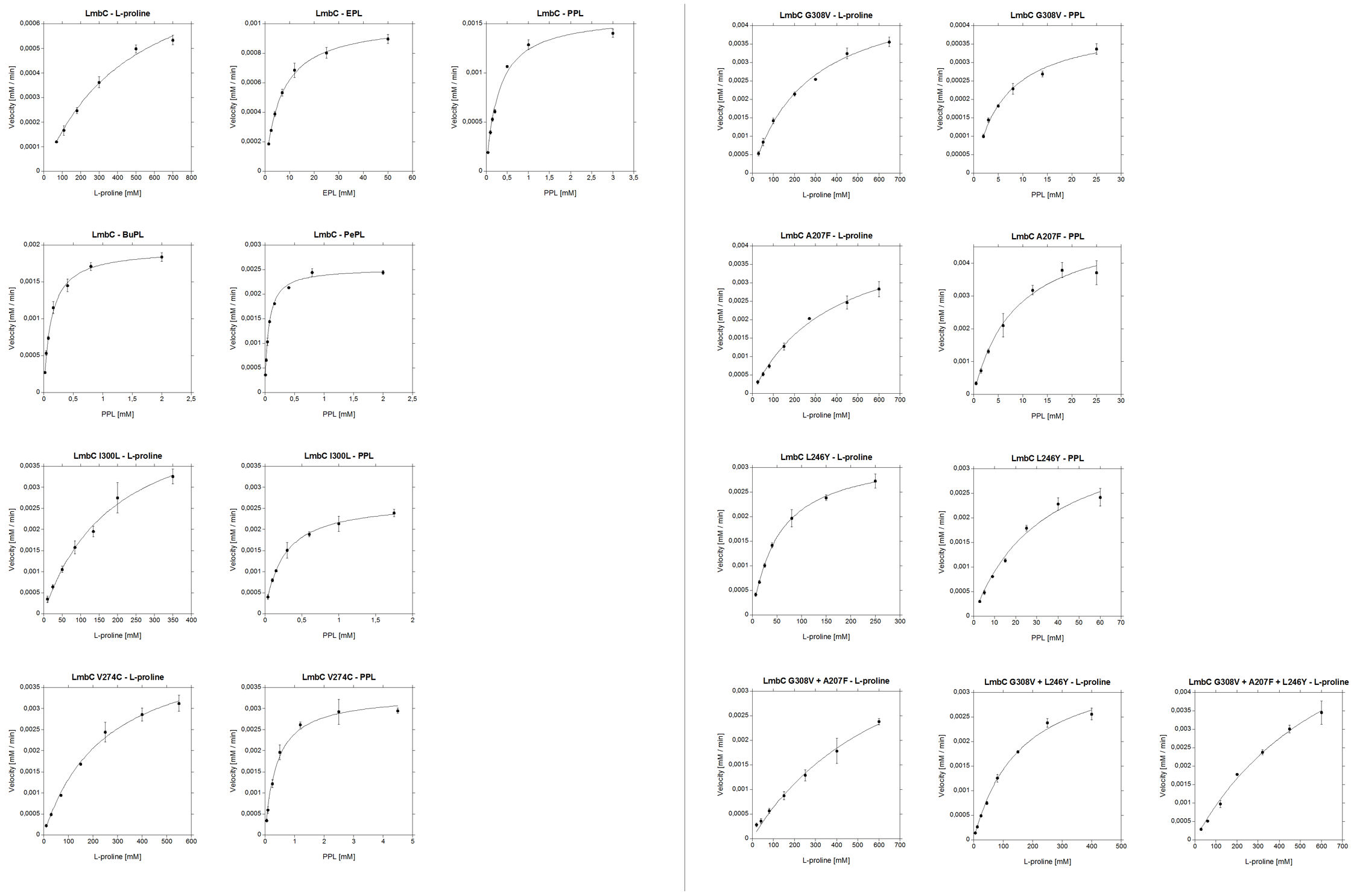

Supplement: S1 Fig — The tested proteins and substrates are written above each graph. All reactions were performed in triplicate. The error bars indicate the standard deviation. The reaction velocity is expressed as the amount of radioactive ATP (mM) produced per minut. Reaction conditions are described in the experimental section. (TIF) [file pone.0189684.s001.tif]

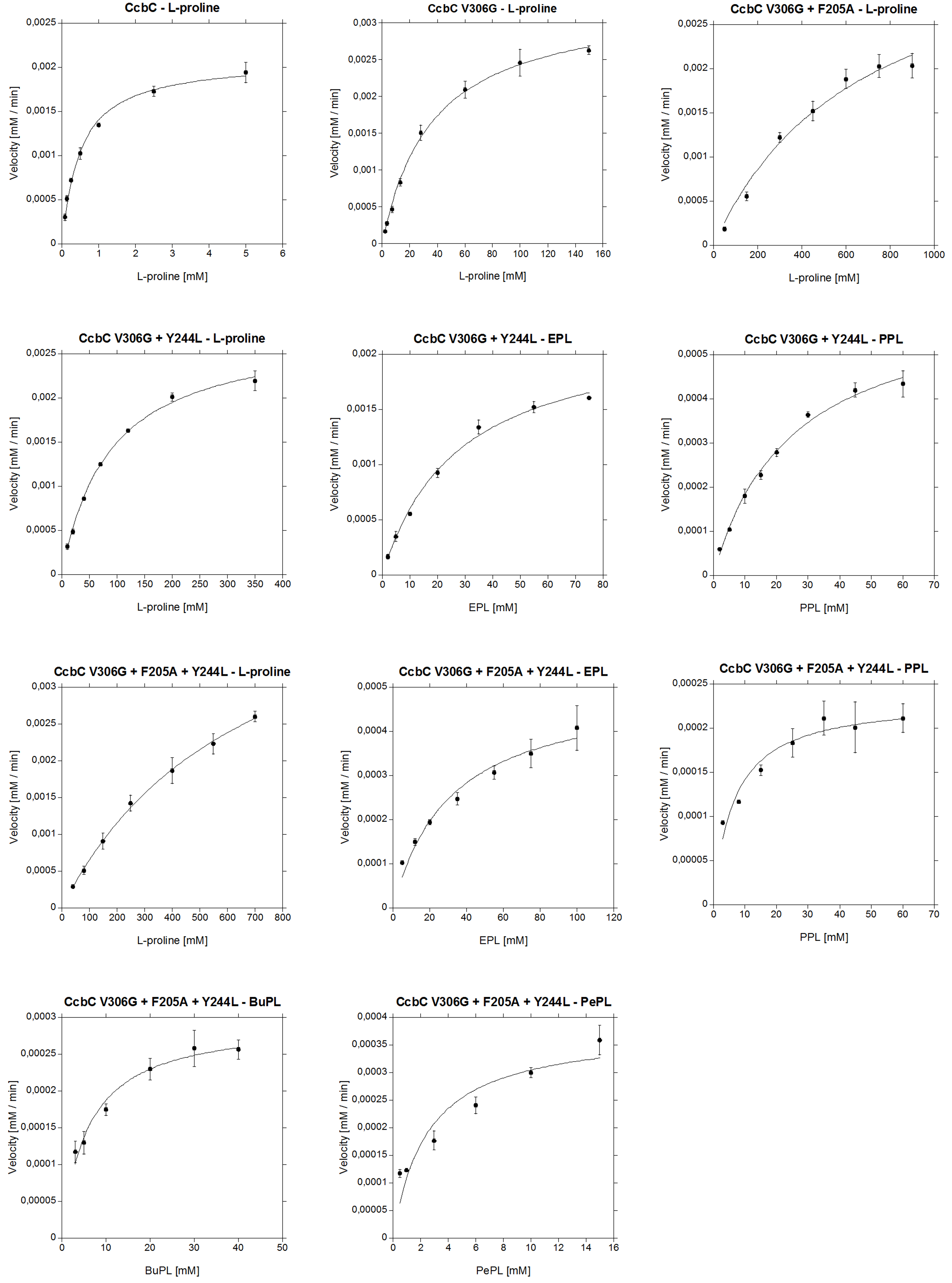

Supplement: S2 Fig — The tested proteins and substrates are written above each graph. All reactions were performed in triplicate. The error bars indicate the standard deviation. The reaction velocity is expressed as the amount of radioactive ATP (mM) produced per minut. Reaction conditions are described in the experimental section. (TIF) [file pone.0189684.s002.tif]

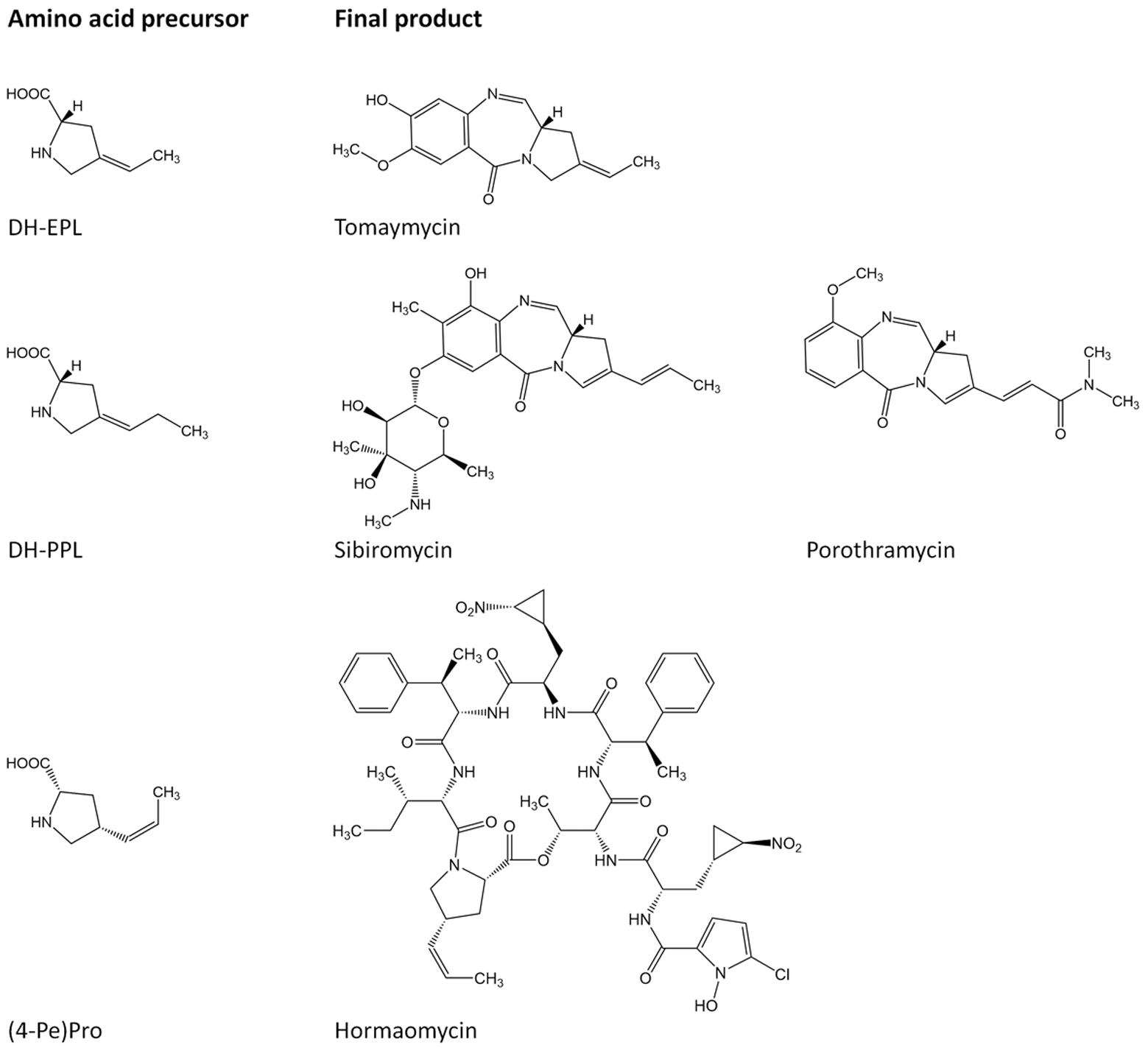

Supplement: S3 Fig — Amino acid precursors activated by appropriate A-domains are in the first column.DH-EPL—4-ethylidene-L-proline, DH-PPL—4-propylidene-L-proline, (4-Pe)Pro—4-propenyl-L-proline. (TIFF) [file pone.0189684.s003.tiff]

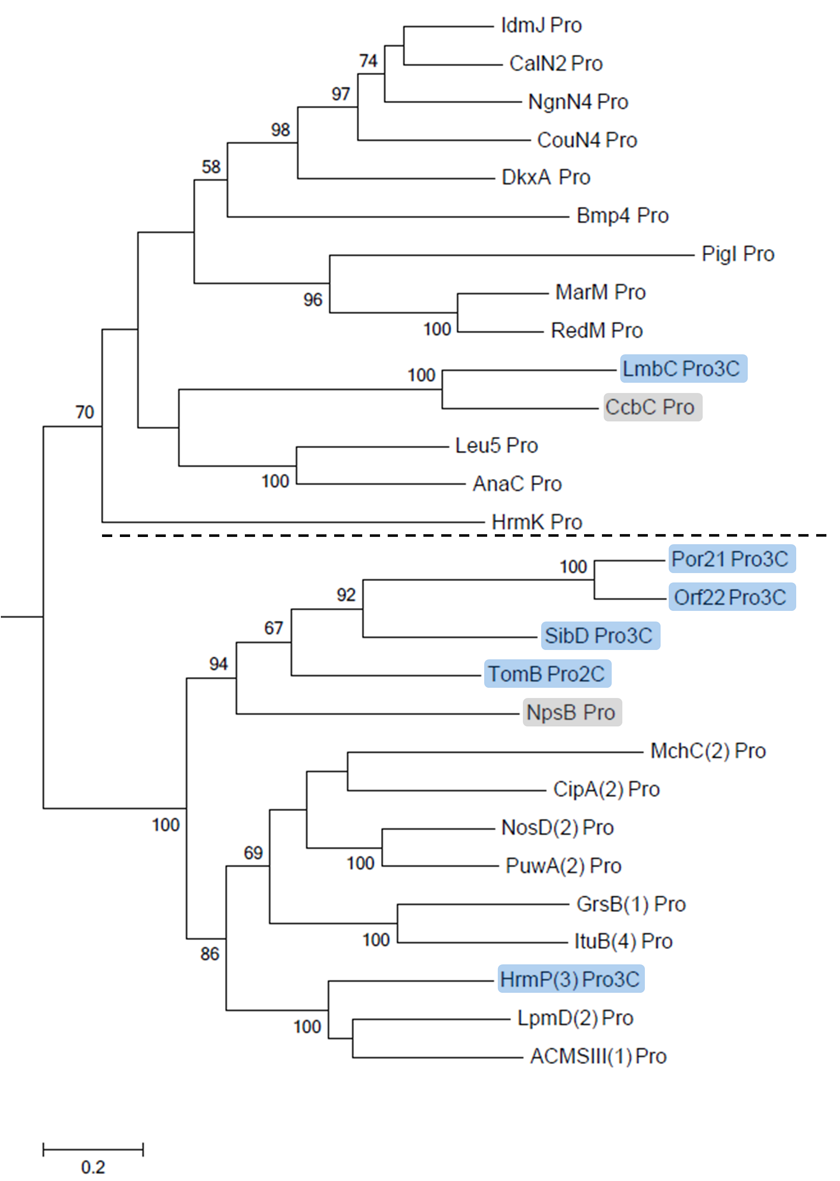

Supplement: S4 Fig — A rooted, maximum likelihood phylogenetic tree was constructed based on the full length amino acid sequences of representative stand-alone A-domains and excised sequences of representative modular NRPS A-domains. Bootstrap values (100 replicates) above 50% are indicated at the nodes. Number in parentheses behind the name of respective NRPS denotes the number of the module in NRPS protein chain, if relevant. The substrate of each A-domain is indicated next to its name. The substrates include L-proline (Pro), L-proline derivatives with two carbon side chain (Pro2C), and L-proline derivatives with three carbon side chain (Pro3C). A-domains specific for Pro2C or Pro3C substrates are highlighted in blue. Their closely related A-domains specific for L-proline are highlighted in grey. The phylogenetic analysis separated A-domains into two clades. Stand-alone A-domains that all, except LmbC, activate L-proline are above the dotted line. Modular NRPS A-domains are below the line, where are the APD activating A-domains split into two branches (Por21, Orf22, SibD, TomB from the biosynthesis of representative PBDs and HrmP(3) from the biosynthesis of hormaomycin). The GenBank accession numbers of stand-alone A-domains are IdmJ–ACN6998.1, CalN2 –AEH42484.1, NgnN4 –AEI59690.1, CouN4 –AAG29789.1, DkxA–CAQ34914.1, Bmp4 –AKJ75110.1, PigI–CAH55654.1, MarM–AHF22853.1, RedM–CAA16182.1, LmbC–ABX00600.1, CcbC–ADB03652.1, Leu5 –ADZ24989, AnaC–ACR33075.1, HrmK–AEH41789.1.The GenBank accession numbers of modular NRPS A-domains are Por21 –AEA29644.1, Orf22 –ABW71853.1, SibD–ACN39727.1, TomB–ACN39015.1, NpsB–CDG76959.1, MchC(2)–CAG29032.1, CipA(2)–AHZ34238.1, NosD(2)–AAF17281.1, PuwA(2)–AIW82277.1, GrsB(1)–BAA06146.1, ItuB(4)–BAB69699.1, HrmP(3)–AEH41794.1, LpmD(2)–AEG64698.1, ACMSIII(1)–CCO61885.1. (TIF) [file pone.0189684.s004.tif]
