# Supplementary material for: Evolution-guided adaptation of an adenylation domain substrate specificity to an unusual amino acid
Source: PLoS One. 2017 Dec 14;12(12):e0189684. doi: 10.1371/journal.pone.0189684 (PMC5730197; doi:10.1371/journal.pone.0189684)
Supplement: S1 Table — (DOCX) [file pone.0189684.s005.docx]

S1 Table. Primers used for site-directed mutagenesis.

| **Mutated gene** | **Coded mutation** | **Primer name** | **Primer sequence (5´ → 3´)** |
| --- | --- | --- | --- |
| *lmbC* | A207F | A207F_for | CGACGTGTCGGTCTTCTCGGTGCTCGGCTCCGC |
|  |  | A207F_rev | GCGGAGCCGAGCACCGAGAAGACCGACACGTCG |
|  | L246Y | L246Y_for | GCCGTCACCGTGTGGTACTCCGTGCCCTATCCG |
|  |  | L246Y_rev | CGGATAGGGCACGGAGTACCACACGGTGACGGC |
|  | V274C | V274C_for | GCCTCGCCACCTTAAGGACGGTCTGCTTCGCCGGTGACGTC |
|  |  | V274C_rev | GACGTCACCGGCGAAGCAGACCGTCCTTAAGGTGGCGAGGC |
|  | I300L | I300L_for | GCCCGGTTCCTCAACCTCTACGGTCCGACCGAGACC |
|  |  | I300L_rev | GGTCTCGGTCGGACCGTAGAGGTTGAGGAACCGGGC |
|  | G308V | G308V_for | CAACATCTACGGTCCGACCGAGACCAACGTCTGTACGTACG |
|  |  | G308V_rev | CGTACGTACAGACGTTGGTCTCGGTCGGACCGTAGATGTTG |
| *ccbC* | F205A | F205A_for | GACGTTTCGGTGGCCAGCCTCTTCGGTGCTGCTCTG |
|  |  | F205A_rev | CAGAGCAGCACCGAAGAGGCTGGCCACCGAAACGTC |
|  | Y244L | Y244L_for | CACGGTGTGGCTCTCGGTACCCTACCCGCTGG |
|  |  | Y244L_rev | CCAGCGGGTAGGGTACCGAGAGCCACACCGTG |
|  | V306G | V306G_for | GAGACGAATGGGTGCACGTTCGAGCAGGTCCTAGCACCGCCGGAC |
|  |  | V306G_rev | GTCCGGCGGTGCTAGGACCTGCTCGAACGTGCACCCATTCGTCTC |
