# Supplementary material for: Evolution-guided adaptation of an adenylation domain substrate specificity to an unusual amino acid
Source: PLoS One. 2017 Dec 14;12(12):e0189684. doi: 10.1371/journal.pone.0189684 (PMC5730197; doi:10.1371/journal.pone.0189684)
Supplement: S2 Table — (DOCX) [file pone.0189684.s006.docx]

S2 Table. Primers used for preparation of the chimeric *ccbC* gene.

| **Primer name** | **Primer sequence (5´ → 3´)** |
| --- | --- |
| CcbC1_for | CCGGAATTCCATATGAATACCTCCACTGTCCG |
| CcbC1_rev | CCGGCTGATGGCCACGCC |
| CcbC2_for | CTCGCACCGCCGGACGAG |
| CcbC2_rev | AACCCAAGCTTACAGCGTGACGTACCG |
| LmbC1_for | GGCGTGGCCATCAGCCGGGCGGGCGCCGTCGCCTT |
| LmbC1_rev | CTCGTCCGGCGGTGCGAGCACGACCTCGTACGTACAGC |
