# Supplementary material for: Evolution-guided adaptation of an adenylation domain substrate specificity to an unusual amino acid
Source: PLoS One. 2017 Dec 14;12(12):e0189684. doi: 10.1371/journal.pone.0189684 (PMC5730197; doi:10.1371/journal.pone.0189684)
Supplement: S3 Table — (DOCX) [file pone.0189684.s007.docx]

S3 Table. Kinetic parameters of LmbC and LmbC mutants for L-proline and PPL substrates.

|  | **L-proline** | | | **PPL** | | |
| --- | --- | --- | --- | --- | --- | --- |
| **Adenylation domain** | **K_m_ [mM]** | **k_cat_ [min^-1^]** | **k_cat_/K_m_  [mM^-1^ min^-1^]** | **K_m_ [mM]** | **k_cat_ [min^-1^]** | **k_cat_/K_m_  [mM^-1^ min^-1^]** |
| LmbC ^[a]^ | 480 ± 70 | 20 ± 1 | 0.042 | 0.28 ± 0.03 | 33 ± 1 | 120 |
| LmbC G308V + A207F | 690 ± 160 | 1.2 ± 0.2 | 0.0018 | NA | NA | NA |
| LmbC G308V + L246Y | 160 ± 20 | 5.7 ± 0.3 | 0.036 | NA | NA | NA |
| LmbC G308V + A207F + L246Y | 730 ± 120 | 1.7 ± 0.2 | 0.0023 | NA | NA | NA |

[a] The previously characterized form, re-measured in the frame of the new experiments.

NA – tested, no detectable activity. PPL - (2*S*,4*R*)-4-propyl-proline. The error values indicate the standard error.
