# Supplementary material for: Evolution-guided adaptation of an adenylation domain substrate specificity to an unusual amino acid
Source: PLoS One. 2017 Dec 14;12(12):e0189684. doi: 10.1371/journal.pone.0189684 (PMC5730197; doi:10.1371/journal.pone.0189684)
Supplement: S4 Table — (DOCX) [file pone.0189684.s008.docx]

S4 Table. Kinetic parameters of the remaining CcbC mutants for various substrates.

| **Adenylation domain** | **Substrate** | **K_m_ [mM]** | **k_cat_ [min^-1^]** | **k_cat_/K_m_ [mM^-1^ min^-1^]** |
| --- | --- | --- | --- | --- |
| CcbC V306G | L-proline | 37 ± 2 | 39 ± 1 | 1 |
| CcbC V306G + F205A | L-proline | 670 ± 180 | 6 ± 0.9 | 0.009 |
| CcbC V306G + Y244L | EPL | 27 ± 3 | 0.15 ± 0.008 | 0.0056 |
| CcbC V306G + F205A + Y244L | EPL | 31 ± 7 | 0.05 ± 0.004 | 0.0015 |
| CcbC V306G + F205A + Y244L | BuPL | 5.8 ± 1 | 0.021 ± 0.001 | 0.0036 |

EPL - (2*S*,4*R*)-4-ethyl-proline and BuPL - (2*S*,4*R*)-4-butyl-proline. The error values indicate the standard error.
